# Brain-heart coupling in tourette syndrome: a precision functional mapping study of discordant siblings

**DOI:** 10.3389/fnetp.2026.1881914

**Published:** 2026-09-23

**Authors:** Sana A. Ali, Damion V. Demeter, Abigail R. Baim, Jonathan Ahern, J. H. Ben Ahn, Sarah E. Chang, Emily M. Koithan, Sujin Park, Benjamin L. Smarr, Deanna J. Greene

**Affiliations:** 1 Department of Cognitive Science, University of California, San Diego, La Jolla, CA, United States; 2 Shiu Chen – Gene Lay Department of Bioengineering, Jacobs School of Engineering, University of California, San Diego, La Jolla, CA, United States; 3 Halicioğlu Data Science Institute, University of California, San Diego, La Jolla, CA, United States

**Keywords:** brain-heart coupling, functional connectivity, individual differences, network physiology, tourette syndrome

## Abstract

Tourette syndrome (TS) is a neurodevelopmental disorder characterized by motor and vocal tics that are modulated by internal physiological state and interoceptive state, including premonitory urges and associated changes in short-timescale parasympathetic variability. Since tic expression is thought to be linked to fluctuations in autonomic state, understanding how brain networks integrate physiological signals may provide insight into the neural mechanisms contributing to tic generation and suppression. In recent years, there has been increasing interest in understanding interactions between the brain and autonomic physiology, given their shared central role in shaping cognition, emotion, and behavior. Methodological advances have enabled the study of brain–heart coupling, yet much of this work has focused on static or region-based approaches, limiting insight into how autonomic fluctuations are integrated within dynamic, large-scale brain networks. Here, we examined time-resolved brain-heart interactions using densely sampled resting-state fMRI data and concurrent pulse oximetry in two sibling pairs (n = 4) discordant for Tourette syndrome (TS). Using two targeted network analyses organized around interoceptive/urge awareness and inhibitory control/action regulation processes, we quantified coupling between pulse-derived RMSSD and between-network functional connectivity using sliding-window analyses and Bayesian linear mixed-effects models. We observed marked individual variability in functional network organization and autonomic measures. The most consistent observation was a pattern of opposing coupling directions between individuals with and without TS: those with TS showed positive associations between pulse-derived RMSSD with connectivity between internal-state and attentional networks, as well as among action-related systems, while unaffected siblings showed positive associations along broader integrative pathways linking internal-state and action-control networks. These preliminary findings generate hypotheses regarding individualized patterns of brain-heart coupling in TS and illustrate the potential value of integrating individualized network mapping with time-resolved physiological measures in clinical populations.

## 1 Introduction

Tourette syndrome (TS) is a neurodevelopmental disorder characterized by unwanted, repetitive motor and vocal tics, affecting approximately 0.3%–0.9% of school-aged children (Knight et al., 2012; DSM, 2013). The underlying neurobiology of TS involves disruptions in cortico-striato-thalamo-cortical circuits, which play a crucial role in integrating motor, sensory, and emotional processing (Albin and Mink, 2006; Haber, 2016). Tic expression can be linked to fluctuations in internal state, including sensations of discomfort that often precede tic onset (premonitory urges) and stress-related changes in arousal, highlighting the role of brain-body interactions in symptom modulation (Leckman et al., 1989; Conelea and Woods, 2008; Ganos et al., 2013). TS is strongly familial and heritable, with first-degree relatives of affected individuals showing elevated rates of tic disorders and related conditions even in the absence of a formal diagnosis (Pauls et al., 2014; Mataix-Cols et al., 2015), suggesting that the neural and physiological features underlying TS liability may vary across family members in ways that are not captured by group-level descriptions.

A longstanding hypothesis holds that the autonomic nervous system (ANS) contributes to the etiology of TS. Tic severity and frequency fluctuate with emotional and physiological state, and these states are themselves indexed by autonomic activity (Thayer and Lane, 2000; Leckman, 2003). This motivates the hypothesis that autonomic function is not a correlate but a candidate contributor to the moment-to-moment expression of tics. However, empirical findings regarding autonomic function in TS have been mixed. Prior cardiovascular studies reported minimal evidence of baseline autonomic abnormalities, suggesting relatively intact resting ANS function in TS (Lake et al., 1977; Goetz et al., 1987). In contrast, other work has identified alterations in sensory habituation, parasympathetic tone, sympathetic reactivity, and stress responsivity in TS, including differences in heart rate dynamics and neuroendocrine markers (van Dijk et al., 1992; Chappell et al., 1994; Hawksley et al., 2015). These inconsistencies likely reflect small sample sizes, methodological heterogeneity, differences in how autonomic function was operationalized, and reliance on group-level analyses that may obscure meaningful individual variation in autonomic-neural relationships. Moreover, prior studies have typically examined autonomic function in isolation, with limited integration of physiological measures and brain function. As a result, it remains unclear how fluctuations in autonomic state (operationalized throughout this study as short-timescale parasympathetic variability as indexed via HRV) relate to the distributed neural systems implicated in TS, underscoring the need for multimodal approaches that jointly characterize brain and autonomic function within individuals.

The relevance of brain-body interactions to TS is further supported by a broader pattern observed across neuropsychiatric and neurodevelopmental disorders. Disruptions in interoceptive processing–the brain’s representation of internal bodily states–have been implicated as a transdiagnostic vulnerability, with evidence suggesting that dysregulated interoceptive predictions may contribute to the generation and maintenance of symptoms across conditions including anxiety, depression, and obsessive-compulsive disorder (Barrett and Simmons, 2015; Khalsa et al., 2018). In TS specifically, premonitory urges have been conceptualized as interoceptive signals arising from abnormal body-state predictions that drive tic expression (Ganos et al., 2015; Rae et al., 2019), raising the possibility that altered coupling between autonomic state and neural systems supporting interoception may be a core feature of TS Symptomatology.

This idea motivates examining brain-heart interactions not merely as a correlate of autonomic function but as a window into the neural mechanisms through which internal physiological signals are translated into tic-related behavior. While interoceptive processing spans multiple bodily systems, the present study focuses specifically on brain-heart interactions, indexed via pulse-derived cardiac measures. Although photoplethysmography (PPG) measures peripheral pulse waves rather than cardiac electrical activity directly, resting-state root mean square of successive differences (RMSSD) derived from PPG has been shown to demonstrate acceptable agreement with ECG-derived RMSSD and is widely used as a noninvasive estimate of cardiac vagal activity (Dewig et al., 2024; Plews et al., 2017). Accordingly, throughout this study we interpret RMSSD as a pulse-derived estimate of short-timescale parasympathetic variability.

Understanding brain-body interactions in TS requires situating them within the literature on brain-heart coupling, which has foundational insights about how autonomic fluctuations are integrated with neural systems across health and disease. The coordination between the central neural system and peripheral physiology is fundamental to adaptive behavior. Converging clinical and experimental evidence demonstrates that cardiovascular, neurological, and psychiatric disorders can disrupt brain–heart interactions, with consequences for perception, cognition, and action (Samuels, 2007; Silvani et al., 2016). Fluctuations in autonomic state influence cognition, emotion, and action, while brain systems dynamically regulate cardiovascular function in response to internal and external demands (Craig, 2002). This bidirectional communication, mediated through afferent and efferent pathways including vagal and spinal mechanisms, plays a key role in information processing, emotional experience, and spontaneous cognition (Azzalini et al., 2019; Chen et al., 2021; Candia-Rivera, 2022; Skora et al., 2022).

Recent work has increasingly emphasized an embodied perspective on brain function, in which cognition and behavior emerge from continuous interactions between neural systems and the body (Chen et al., 2021; Quigley et al., 2021). Within this context, brain-heart interactions have been studied using approaches such as heartbeat-evoked responses–neural responses time locked to the cardiac cycle measured with EEG or fMRI–and measures of coupling between autonomic dynamics and neural activity, including correlations between heart rate variability (HRV) and spontaneous brain fluctuations (Park and Blanke, 2019; Candia-Rivera et al., 2021; Coll et al., 2021). A common approach to quantifying autonomic state is through HRV, which has been linked to emotional regulation, cognitive control, and adaptive behavioral responding (Lane et al., 2009; Thayer et al., 2009). Resting-state functional magnetic resonance imaging (fMRI) studies further demonstrate that spontaneous fluctuations in HRV covary with functional connectivity (FC) – the statistical coupling of neural activity between brain regions or networks–across distributed brain systems, particularly within insula, cingulate, and medial prefrontal regions (Chang et al., 2013; Sakaki et al., 2016). However, these approaches have largely characterized relationships between cardiac signals and localized brain regions or group-averaged connectivity patterns, providing limited insight into how autonomic fluctuations are integrated across large-scale functional networks at the level of individual people–a level of organization that may be critical for understanding individual differences in cognition and behavior (Bressler and Menon, 2010; Smith et al., 2011; 2011; Park and Friston, 2013; Laumann et al., 2015; Gordon et al., 2017b).

This limitation is particularly consequential for understanding TS, where individual differences in network organization and autonomic reactivity may be obscured by traditional group-averaging approaches. Reliable estimation of individualized large-scale network organization requires sufficient signal-to-noise ratio at the level of individual participants, which is a challenge that conventional single-session fMRI designs are underpowered to meet. Precision functional mapping (PFM) addresses this gap by acquiring densely sampled within-participant fMRI data across multiple sessions, boosting signal-to-noise ratio to enable stable individual-specific network estimates that reveal substantial inter-individual variability–particularly in higher-order association systems–that conventional approaches obscure (Laumann et al., 2015; Gordon et al., 2017b; Dworetsky et al., 2024).

Grounded in network physiology principles and leveraging PFM to characterize individualized network organization, the present study treats the brain and ANS as dynamically coupled subsystems whose coordinated interactions underlie adaptive physiological function (Bashan et al., 2012; Bartsch et al., 2015; Ivanov et al., 2016). We examined brain-heart interactions at the level of large-scale functional networks in a pilot study of two individuals with TS and their unaffected siblings, targeting two core mechanistic dimensions of TS symptomatology. We hypothesized that autonomic state would be systematically coupled with FC across both analyses, and that the pattern of brain-heart coupling would differ between individuals with TS and their unaffected siblings. Notably, the discordant sibling design provides a genetically and environmentally controlled comparison, allowing us to examine whether observed brain-heart coupling differences reflect disorder-specific neural organization beyond shared familial factors.

The first analysis (Target Analysis 1) targeted interoceptive and urge awareness processes, examining network connections among the default mode (DMN), salience (SAL), ventral attention/language (VAN/LANG), and somato-cognitive action (SCAN) networks. These networks were selected because they collectively support the internal-state and attentional processes most relevant to premonitory urge generation and awareness: DMN supports internal state representation and self-referential processing (Buckner et al., 2008) (see also Beissner et al., 2013 on parasympathetically-dominant central autonomic nuclei associated with DMN) and is activated during tic suppression (Peterson et al., 1998; Ganos et al., 2014; van der Salm et al., 2018; Greene et al., 2025); SAL is involved in detecting interoceptive signals and mediating autonomic responses (Menon and Uddin, 2010); VAN/LANG supports attentional reorienting to salient internal and external events (Corbetta and Shulman, 2002; Corbetta et al., 2008) as well as language processing (Fedorenko et al., 2024); and SCAN integrates cognitive and somatic signals to shape motor output with functional connections to insula regions implicated in processing interoceptive signals, suggesting a role in integrating bodily state information with action planning (Worbe et al., 2015; Gordon et al., 2023). Additional evidence suggests that DMN and SAL are differentially dominated by parasympathetically- and sympathetically-associated central autonomic nuclei, respectively (Beissner et al., 2013), and that vagally-mediated cardiac-autonomic outflow is better characterized as distributed across a broader central autonomic network than localized to any single region (Valenza et al., 2019), further motivating the examination of coupling between these networks and cardio-autonomic oscillations in TS. Together, these networks capture the pathway from autonomic fluctuation to urge awareness to motor expression central to tic generation in TS. We predicted that TS-specific coupling would be most evident in connections linking internal-state and attentional systems within Target Analysis 1, reflecting a stronger association between autonomic state and coordination of the urge-related attentional network in TS.

The second analysis (Target Analysis 2) targeted inhibitory control and action regulation processes, examining network connections among the frontoparietal network (FPN), action mode network (AMN), SCAN, and DMN. FPN supports top-down regulatory control over motor output and goal-directed behavior (Dosenbach et al., 2007); AMN supports sustained goal maintenance, arousal regulation, action planning, and sympathetic drive for physiological control, including functional connectivity to the adrenal medulla and other peripheral autonomic structures which are relevant to the autonomic states that modulate tic expression (Dosenbach et al., 2025); and SCAN serves as a bridge between action-mode circuitry and effector-specific motor systems, with strong FC to AMN for whole-body action planning and physiological control (Gordon et al., 2023). DMN and SCAN was included in this analysis as cross-analysis anchors. DMN was included given its involvement in internally generated states that may interact with top-down inhibitory control systems during tic suppression and given that AMN and DMN may counterbalance each other as opposing functional modes of brain organization (Dosenbach et al., 2025). SCAN was included given its role as a bridge between action-mode and effector-specific motor systems, connecting the two analyses at the level of somatic-cognitive action integration. Together, these networks capture the regulatory pathway through which cognitive control systems modulate motor output in the context of fluctuating autonomic state. Within Target Analysis 2, we predicted that TS-specific coupling would be most evident in connections between action-mode and somato-cognitive systems, reflecting dysregulated coupling between autonomic state and inhibitory control circuitry.

## 2 Methods

Briefly, resting-state fMRI and PPG were acquired concurrently in participants with Tourette syndrome and their unaffected siblings. Each modality was preprocessed independently to generate individualized functional network maps and beat-to-beat cardiac intervals. Sliding-window functional connectivity and RMSSD measures were then derived and used to model within-subject brain-heart coupling across two targeted sets of network pairs. Sections 2.1–2.7 describe each step of this workflow in detail.

### 2.1 Participants

Participants consisted of two discordant sibling pairs recruited from an ongoing pediatric PFM study at UC San Diego, approved by the Institutional Review Board (#801399). Participants were recruited from the broader San Diego community and Rady Children’s Hospital. The sample consisted of two sibling pairs, each pair comprising one child with a formal diagnosis of Tourette’s Syndrome (TS) and their unaffected sibling (US) (Table 1). Participant psychiatric diagnoses were confirmed using the Kiddie Schedule for Affective Disorders and Schizophrenia (KSADS (Kaufman et al., 1997)) by a trained rater. In one of the sibling pairs (PT105, PT106), both children met criteria for Attention Deficit Hyperactivity Disorder (ADHD). In TS participants, tic severity was assessed using the Yale Global Tic Severity Scale (YGTSS (Leckman et al., 1989)).

**TABLE 1:** Participant demographic, resting-state fMRI, PPG, and clinical information.

Subject ID	Age	Sex	Number of fMRI scans	Low motion fMRI data (min)	Total usable PPG (min)	Race	Ethnicity	Psychiatric diagnoses
PT105	12	F	6	116.72	35.11	NR	Hispanic	TS, ADHD, GAD, OCD
PT106	13	F	4	122.94	64.81	NR	Hispanic	ADHD, anxiety
PT110	10	M	4	47	37.81	Asian	NH	TS
PT111	13	F	4	95.56	48.61	Asian	NH	None

NR, Not reported. NH, Non-Hispanic. TS, Tourette Syndrome. ADHD, Attention Deficit/Hyperactivity Disorder. GAD, Generalized Anxiety Disorder. PPG, photoplethysmography.

PPG acquisition and quality summary for resting-state runs included in analyses. Values are summarized across quality-control-passing resting-state PPG runs for each participant.

### 2.2 Neuroimaging acquisition

Participants were scanned at the University of California, San Diego, on a Siemens Prisma 3T MRI scanner with a 32-channel head coil. Participant fMRI data were collected over 4 to 6 sessions each (Table 1). At least one T1-weighted structural MPRAGE sequence (TR = 2500 m,TE = 2.9 m, FOV = 256 × 256, voxel resolution = 1x1x1mm) and one T2-weighted structural image with turbo spin echo sequence (TR = 3200 m, TE = 564 m, FOV = 256 × 256, voxel resolution=1x1x1mm) were collected in each visit. Up to six 6-min resting-state fMRI runs, collected with a five-echo blood oxygen level dependent (BOLD) contrast sensitive gradient echo-planar sequence (TR = 1761 m, multiband 6 acceleration, TE1: 14.20 m, TE2: 38.93 m, TE3: 63.66 m, TE4: 88.39 m, and TE5: 113.12 m, flip angle = 68°, resolution = 2.0 mm isotropic) were collected per visit. During the eyes-open resting-state fMRI scans, TS participants were instructed that it was acceptable to tic as needed while trying to remain as still as possible when not ticcing. Head motion was monitored during scans using the Framewise Integrated Real-time MRI Monitor (FIRMM) software (Dosenbach et al., 2017), allowing scan staff to coach participants and pause acquisition when motion was excessive. Tic events themselves were not independently annotated during scanning: see Discussion. Concurrent PPG was recorded during each run, acquisition details are provided in Section 2.4.

### 2.3 Neuroimaging data processing

MRI data were preprocessed in-house using the DCAN-Labs abcd-hcp-pipeline (Sturgeon et al., 2021), as well as FMRIB Software Library, Freesurfer, Connectome Workbench commands, and custom MATLAB scripts. Multi-echo fMRI data were processed using NOise Reduction with DIstribution Corrected (NORDIC) thermal denoising (Moeller et al., 2021) and Tedana (DuPre et al., 2021) for denoising and optimal combination of multi-echo data. The DCAN-Labs abcd-hcp-pipeline follows the primary steps of the Human Connectome minimal preprocessing pipeline (Glasser et al., 2013) and is then followed by additional resting-state functional connectivity-specific preprocessing steps informed by common best practices (Power et al., 2012; 2014; Hallquist et al., 2013). The FC steps included: (1) de-meaning and de-trending of data; (2) general linear model “denoising” of signal related to white matter, cerebral spinal fluid, whole brain (global) signal, and six directions of motion plus their derivatives; (3) temporal band-pass filtering (0.008 Hz < f < 0.09 Hz); (4) respiratory motion filtering (5) and motion censoring, which excluded frames exceeding a framewise displacement (FD) of 0.2 mm for PT106, PT110, PT111 and 0.3 mm for PT105. FD thresholds for each individual were selected by visual inspection of FD traces to determine the floor for each individual as previously recommended (Power et al., 2014; Power, 2017). Additionally, low-motion frames were retained only in contiguous sets of at least 5 frames for PT105, PT106, and PT111, and at least 3 frames for PT110, determined through visual inspection of FD traces consistent with the individualized thresholding approach described above. Registration steps and denoising were each done in a single pass to mitigate the reintroduction of noise (Lindquist et al., 2019). All resting-state fMRI data were then mapped to an MNI-transformed midthickness 32k fs_LR surface mesh (Van Essen et al., 2012).

### 2.4 Physiological data processing

Autonomic state was indexed using PPG, a non-invasive measure of peripheral cardiovascular dynamics acquired concurrently with resting-state fMRI, from which HRV and related autonomic metrics were derived. PPG was acquired while participants rested in a supine position in the MRI scanner using a wireless finger-clip pulse oximeter attached to the participant’s left index finger, sampling blood oxygen saturation every 4.99 m (∼200 Hz) this sampling rate provides ample temporal resolution for beat-to-beat interval detection and pulse-peak localization for pulse-derived RMSSD, exceeding the approximately 100 Hz generally recommended for robust pulse rate variability analyses (Burma et al., 2024). Because cardiac dynamics were measured via PPG rather than ECG, RMSSD values reported here reflect pulse-derived rather than strictly electrocardiographic vagal indices; we therefore refer to this measure as pulse-derived RMSSD throughout (see Section 4 for discussion of this distinction).

PPG recordings acquired during resting-state fMRI were processed using in-house Python scripts (v3.9.7; SciPy). Signals were bandpass filtered (0.5–5 Hz) using a third-order zero-phase Butterworth filter applied via forward-backward filtering to isolate cardiac pulsatility while minimizing low-frequency drift and high-frequency noise (Charlton et al., 2022)). Cardiac peaks were identified using SciPy’s find_peaks function, with a minimum peak-to-peak separation of 0.4s (corresponding to a physiological maximum of 150 bpm, appropriate for resting-state data) and a prominence threshold set to 0.5 times the within-run standard deviation of the filtered signal, selected empirically and validated through visual inspection of representative waveforms. Inter-beat-intervals (IBIs) were calculated as the temporal difference between successive detected peaks. Intervals shorter than 300 m or longer than 2000 m were considered physiologically implausible and excluded, consistent with standard HRV processing guidelines (Shaffer and Ginsberg, 2017). No additional interpolation-based ectopic-beat correction was applied to IBIs falling within these physiological bounds. Instead, signal quality and peak detection were assessed through visual inspection of the PPG waveforms, and runs with prolonged signal dropout or visually apparent clipping were excluded from further analysis.

HRV was quantified using the root mean square of successive differences (RMSSD), computed from the cleaned IBI series for each run separately as the primary index of short-timescale parasympathetic variability (Shaffer and Ginsberg, 2017). Mean HR was also derived from the cleaned IBI series and retained as a covariate in subsequent analyses to account for baseline differences in cardiac rate that could confound HRV-based indices of autonomic variability. Time windows for brain-heart coupling analyses were defined in TR units and cropped to the fMRI acquisition grid, with window boundaries converted to seconds to extract the corresponding IBI values from the PPG timeseries at its native sampling rate. For each window, RMSSD was computed from the extracted IBIs and paired with the FC estimate derived from the fMRI frames in the same window. Only windows fully covered by the PPG recording were retained for analysis.

### 2.5 Vertex-wise individual-specific network identification

Individual-specific functional networks were identified using the Infomap community detection method (Rosvall and Bergstrom, 2008), adopted by the methodology described in (Gordon et al., 2017b). To measure FC among all vertices on the cortical surface, we first computed pairwise Pearson r correlations among the BOLD time series across all cortical vertices, generating a correlation matrix of dimensions 59,412 × 59,412. This matrix underwent thresholding across a range of edge densities spanning from 0.1% to 5%. For each threshold, community assignments were produced using the Infomap algorithm. Putative functional network identities were assigned to each community by comparing with independent group-derived template networks through Jaccard index (spatial overlap) (Gordon et al., 2017a).

For every threshold, each community was compared to each template and assignments were given to the best match if the Jaccard index was at least 0.1 (lower overlap disregarded to prevent assignments to poorly fitting matches). To consolidate assignments across sparsity thresholds, a consensus network assignment was derived by taking the network identity of a vertex at the sparsest threshold where it was successfully assigned. Each vertex was assigned to one of 11 canonical functional networks, including the auditory (AUD), cingulo-opercular/action-mode (AMN), dorsal attention (DAN), default mode (DMN), frontoparietal network (FPN), somatomotor face (SM-face), somatomotor foot (SM-foot), somatomotor hand (SM-hand), visual (VIS), salience (SAL), and ventral attention/language (VAN/LANG). The somato-cognitive action network (SCAN), was manually identified in each individual using a seed-map approach (as in (Ali et al., 2026) and based on the inter-rater consensus of two raters (authors SA and SP).

### 2.6 Brain-heart coupling analysis

To examine associations between autonomic state and functional brain network organization within individuals, physiological and resting-state functional connectivity measures were quantified using a sliding window approach (Figure 1) applied separately within each participant (Hutchison et al., 2013; Allen et al., 2014). Analyses were restricted to resting-state runs that satisfied motion and physiological quality-control criteria–specifically DCAN FD-based frame censoring (Sturgeon et al., 2021) and successful PPG peak detection–resulting in retention of 69 of 70 rest runs (98.6%), contributing an average of 34.5 windows per participant (range: 26–48) across all sessions. Because concurrent pulse oximetry recordings were shorter than the full fMRI run duration, only windows fully contained within the valid physiological recording period were included in brain-heart coupling analyses.

**FIGURE 1:**
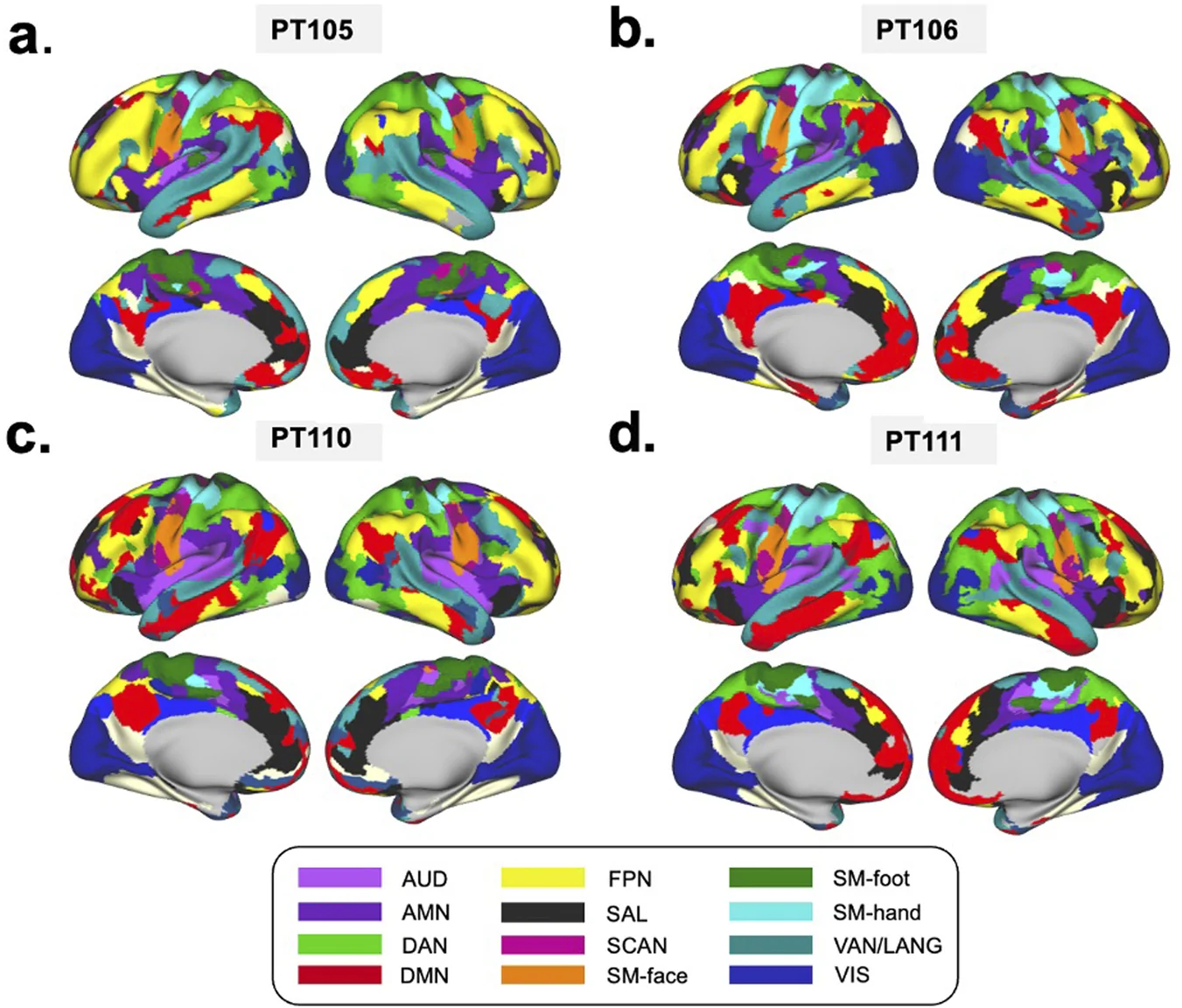
Brain-heart coupling sliding window pipeline. **(a)** Example of sliding-window FC estimation of one participant (PT106). Overlapping windows (dashed lines; 90-s with 45-s steps) are used to compute the Pearson correlation of FC between the mean time series of two network pairs. **(b)** Example of autonomic state estimation using inter-beat intervals (IBIs), from which HRV (RMSSD) is computed within the same windows.

For each resting-state run, network time series were computed as the mean BOLD signal across all cortical vertices assigned to each network, using the individualized functional networks derived from the PFM procedure described in Section 2.5. Analyses were organized around two theoretically motivated network analyses. Target Analysis 1 targeted interoceptive and urge awareness processes, examining connections among the DMN, VAN, SAL, and SCAN networks. Target Analysis 2 targeted inhibitory control and action regulation processes, examining connections among the FPN, AMN, SCAN, and DMN networks. The SCAN and DMN appeared in both analyses, given their theoretical relevance to both interoceptive and regulatory processes.

Dynamic functional connectivity was estimated using overlapping 90-s windows stepped every 45 s. To assess the stability and feasibility of coupling estimates, split-half reliability analyses were conducted across runs for candidate window lengths (90 s, 120 s, and 150 s). Connection-wise RMSSD-connectivity profiles were compared across data splits, and window lengths were evaluated for reliability and data availability to inform the selection of the primary analysis window. Within each window, Pearson correlations between network mean time series were computed and Fisher z-transformed to normalize the distribution of connectivity estimates (Cohen and Cohen, 2003), yielding window-level estimates of between-network functional connectivity for each network pair. Autonomic state was indexed using windowed RMSSD derived from pulse oximetry recordings (Shaffer and Ginsberg, 2017), with mean HR retained as a covariate. Windows were included only if they contained at least 20 valid IBIs (Munoz et al., 2015); observed range: 104–162 IBIs per window; mean = 134).

Within each participant, associations between RMSSD and between-network FC were quantified separately, yielding subject-specific RMSSD-connectivity slopes. These slopes reflect the extent to which within-subject fluctuations in autonomic variability were associated with concurrent changes in coupling between network pairs. These slopes were subsequently entered into participant-level Bayesian linear mixed-effects models to evaluate differences between TS and sibling participants, as described in Section 2.7.

### 2.7 Statistical modeling

To model the relationship between autonomic state and FC, Bayesian linear mixed effects models were fit separately for each between-network connectivity pair and for TS and sibling participants independently using the brms package in R (Bürkner, 2017). This approach is well-suited for the present dataset–a small number of independent participants each contributing many repeated observations across windows and session–allowing estimation of within-subject effects with appropriate uncertainty quantification given our sample size fitting models separately for TS and siblings allowed estimation of participant specific within-subject RMSSD-connectivity associations, which is the primary inferential target of the dual-analysis approach. Network pairs examined included all six connections within Target Analysis 1 (DMN-VAN, DMN-SAL, DMN-SCAN, VAN-SAL, SCAN-VAN, SCAN-SAL) and six pairs within Target Analysis 2 (DMN-FPN, FPN-SCAN, FPN-AMN, DMN-AMN, SCAN-AMN, DMN-SCAN).

FC was modeled as a function of RMSSD using a within-between decomposition, in which the RMSSD predictor was partitioned into within-subject (deviation from subject mean) and between-subject (subject mean) components. This decomposition enables separation of within-subject fluctuations in autonomic state from between-subject differences in average physiology. All predictors were standardized by their pooled standard deviations to facilitate comparison across models. Models included fixed effects for within-subject RMSSD, between-subject RMSSD, mean HR, and number of usable frames per window. A random intercept and an uncorrelated random slope for the within-subject RMSSD effect were included for subject (specified as 1 + predictor_within_z | subject_id). The uncorrelated rather than correlated random effects structure was chosen because the small number of participants per analysis (N = 2) renders the correlated structure unidentifiable. A Student’s t likelihood was specified to accommodate potential deviations from normality in connectivity estimates. Weakly informative priors were specified for all parameters: β ∼ Normal (0, 1), intercept ∼ Normal (0, 2), variance components ∼ Exponential (1), and degrees of freedom ν ∼ Gamma (2, 0.1). The prior on ν was chosen to allow heavier tails than a Gaussian while remaining weakly informative, providing robustness to outlying connectivity estimates without strongly constraining the likelihood. Posterior distributions were estimated using the Markov Chain Monte Carlo (MCMC) sampling with four chains of 4,000 iterations each (2,000 warmup). Convergence was assessed via the R̂ statistic and visual inspection of trace plots; model adequacy was evaluated using posterior predictive checks.

The primary parameter of interest was the within-subject RMSSD slope (β_within_), which quantifies the extent to which fluctuations in autonomic state are associated with concurrent changes in between-network FC within individuals. For each model, posterior medians, 95% credible intervals, and two posterior probability summaries were reported: P (β_within_ > 0), the posterior probability that the within-subject RMSSD slope is positive, reflecting the direction and strength of the autonomic-connectivity association; and P (β_real_ > β_null_), the posterior probability that the observed slope exceeded the slope estimated from shuffled null models, reflecting coupling above chance temporal alignment. To construct null distributions, RMSSD values were circularly shifted across windows within each subject and run, disrupting temporal correspondence between autonomic and neural measures while preserving the observed physiological values, their distribution, and their within-run temporal autocorrelation structure. Null models were fit using identical model specifications as the observed models. We emphasize that these models estimate within-subject associations between RMSSD and connectivity; given only two participants per group, the between groups comparison (TS vs. siblings) should be interpreted as descriptive contrasts between the specific individuals studied rather than inferential tests of group-level of diagnostic effects.

## 3 Results

### 3.1 Individual-specific functional brain networks reveal distinctive network architecture

Resting-state FC data were used to define participant-specific functional brain networks. Each participant displayed a network organization consisting of 12 canonical functional networks, including the default mode (DMN), somatomotor hand (SM-hand), somatomotor face (SM-face), somatomotor foot (SM-foot), cingulo-opercular/action-mode (AMN), somato-cognitive action (SCAN), auditory (AUD), visual (VIS), salience (SAL), language (LANG), dorsal attention (DAN), and frontoparietal networks (FPN) (Figure 2). While individuals shared broad features of functional organization, each had a unique network topography, as expected with PFM (Gordon et al., 2017b).

**FIGURE 2:**
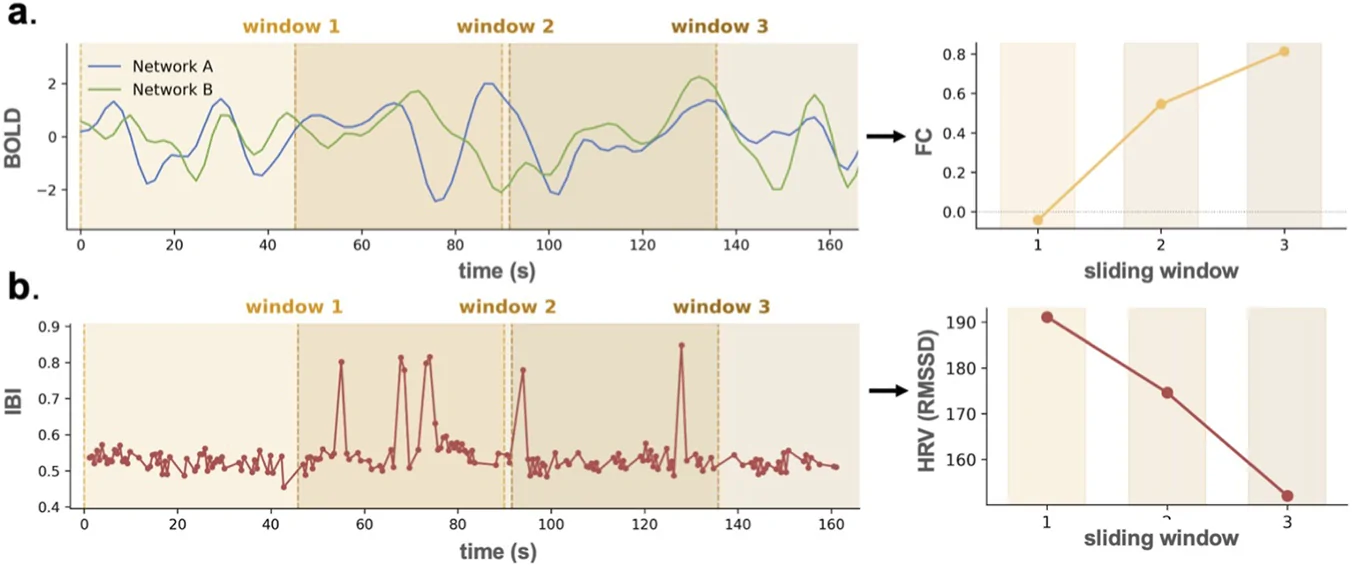
Individualized cortical network maps show individual-level features of large-scale functional brain networks. Panels **(a,c)** depict the two participants with Tourette syndrome (PT105, PT110), and panels **(b,d)** depict their unaffected siblings (PT106, PT111). For each participant, lateral and medial views of both hemispheres are displayed. Colors indicate individualized network assignments across the cortical surface. Abbreviations: Abbreviations: AUD, auditory; AMN, action- mode network; DAN, dorsal attention; DMN, default-mode; FPN, frontoparietal; SAL, salience; SCAN, somato cognitive action; SMF, somatomotor face; SMFoot, somatomotor foot; SMH, somatomotor hand; VAN/LANG, ventral attention/language; VIS, visual.

### 3.2 Physiological measures show within- and between-subject variability

HR and HRV (assessed using RMSSD) varied both across runs within each participant and across participants (Figure 3). Mean RMSSD ranged from 140.5 to 323.8 m across individual runs, with participant-level means spanning 174.2–247.4 m; within-subject run-to-run variability in RMSSD averaged 31.8 m (range: 18.9–45.7 m across participants). Mean HR ranged from 78.2 to 95.8 bpm across participants (individual run range: 71.3–106.3 bpm). These RMSSD values are consistent with pediatric normative ranges, in which children exhibit substantially higher vagal tone and respiratory sinus arrhythmia amplitude than adults (Umetani et al., 1998).

**FIGURE 3:**
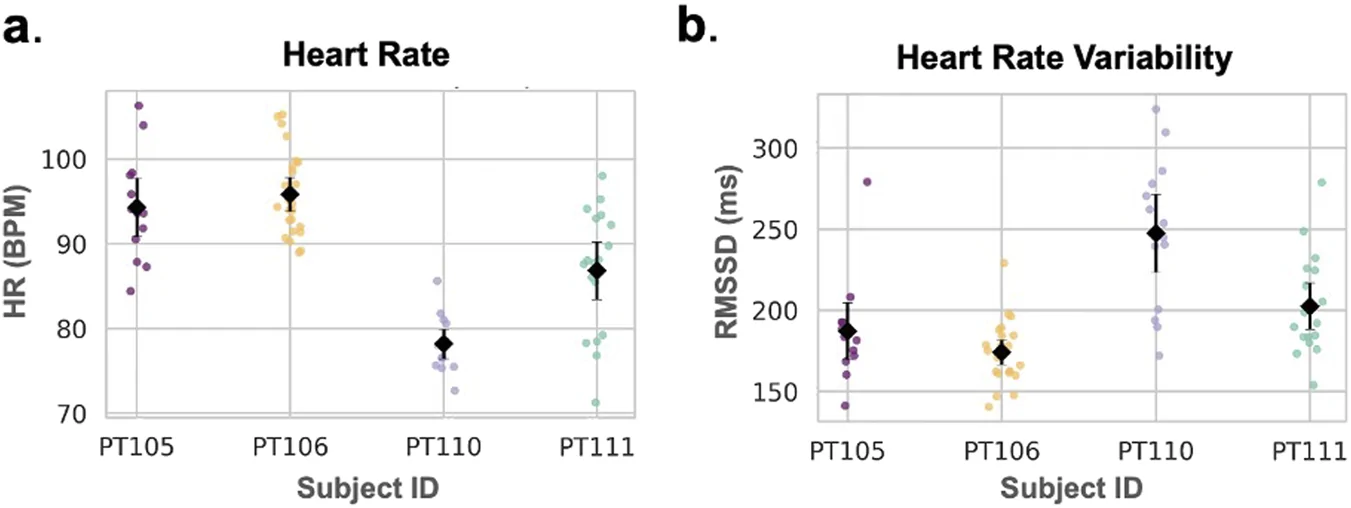
Run-level heart rate and RMSSD by subject. Each point represents one resting-state fMRI run. Black diamonds indicate the subject mean and error bars indicate the 95% confidence interval across runs. The left panel shows run-level mean heart rate (HR; beats per minute), and the right panel shows run-level root mean square of successive differences (RMSSD; ms). Abbreviations: HR, heart rate; RMSSD, root mean square of successive differences.

Run-level summaries confirmed differences in both mean HR and RMSSD across individuals, as well as consistent run-to-run fluctuations within individuals. Notably, the dominant pattern of physiological variation appeared familial rather than diagnostic: Pair 1 (PT105/PT106) showed higher HR and lower RMSSD than Pair 2 (PT110/PT111) regardless of TS status, while within Pair 2, PT110 showed higher RMSSD than sibling PT111, and PT105 and PT106 were very similar to each other. Thus, in our sample, physiological measures followed familial or developmental factors shared within sibling pairs rather than TS diagnosis. The within- and between-subject dynamic range in pulse-derived RMSSD was nevertheless sufficient to support time-resolved brain–heart coupling analyses.

### 3.3 Split-half analyses support 90-s windows as the most reliable timescale

Split-half reliability of the RMSSD-network FC coupling beta estimates varied considerably across candidate window lengths. Reliability was assessed by splitting available runs into two-halves and correlating connection-wise RMSSD–FC slope profiles across splits–that is, examining whether the same network connections showed consistent directional associations with RMSSD in each data half. The 90-s window yielded the highest mean reliability (r = 0.64), outperforming the 150-s (r = 0.20) and 120-s (r = 0.09) windows. Windows shorter than 90 s were not evaluated because they would yield insufficient inter-beat intervals per window to support stable RMSSD estimation, given the 20 IBI minimum threshold established in Section 2.7. This counterintuitive pattern, longer windows showing less reliability, likely reflects the trade-off between window duration and the number of available windows in a dense-sampling, small-N design: longer windows reduce the total number of observations, which can destabilize split-half estimates despite providing more data per window. The 90-s window, therefore, offered the best balance between temporal resolution and estimate stability given the available data, and was selected for all primary analyses. This overlapping-window approach (45-s step) supported reliable estimation of both autonomic variability and FC across participants.

### 3.4 Opposing autonomic coupling directions across interoceptive networks in TS and siblings

Bayesian models fit separately for TS and sibling participants show preliminary opposing directional tendencies in RMSSD-FC coupling across multiple network connections in Target Analysis 1 (Figure 4; Supplementary Table S1). Among the participants with TS, the clearest positive directional tendency was observed at the DMN-VAN/LANG connection, where RMSSD showed a positive directional relationship with FC in TS participants (β = 0.27, 95% CrI [−0.60, 1.01], P (β > 0) = 0.83) but not in siblings (β = −0.02, P (β > 0) = 0.43). A similar pattern, with positive directional tendencies in the participants with TS and negative tendencies in their unaffected siblings, was observed at the SCAN-SAL connection (TS: P (β > 0) = 0.74; Sibling: P (β > 0) = 0.32), which also showed the most consistent real-over-null separation in TS across Target Analysis 1 connections (P (β_real_ > β_null_) = 0.69). A complementary but directionally reversed pattern emerged at the DMN-SCAN and SCAN-VAN connections. Siblings showed positive tendencies at both (P (β > 0) = 0.87 and 0.68, respectively), while TS participants showed negative slopes (P (β > 0) = 0.15 and 0.18, respectively), suggesting that, within this sample, higher pulse-derived RMSSD may be associated with reduced coupling at these connections in TS. DMN-SCAN exhibited the largest directional difference observed in this sample, with TS real-data slopes falling predominantly below null (P (β_real_ > β_null_) = 0.31) while siblings trended above null (P (β_real_ > β_null_) = 0.49). VAN-SAL and DMN-SAL showed near-chance posteriors in both TS and siblings participants and no meaningful differentiation between them. Taken together, Target Analysis 1identified different directional patterns of RMSSD-FC interoceptive network coupling in qualitatively different ways in the present sample: the TS participants showed a positive RMSSD signature at the DMN-VAN and SCAN-SAL connections, with opposing negative tendencies at DMN-SCAN and SCAN-VAN that were not observed in the siblings. All credible intervals crossed zero, reflecting the limited diagnostic effect based on the current data and sample size, and results should be interpreted as directional trends rather than definitive estimates.

**FIGURE 4:**
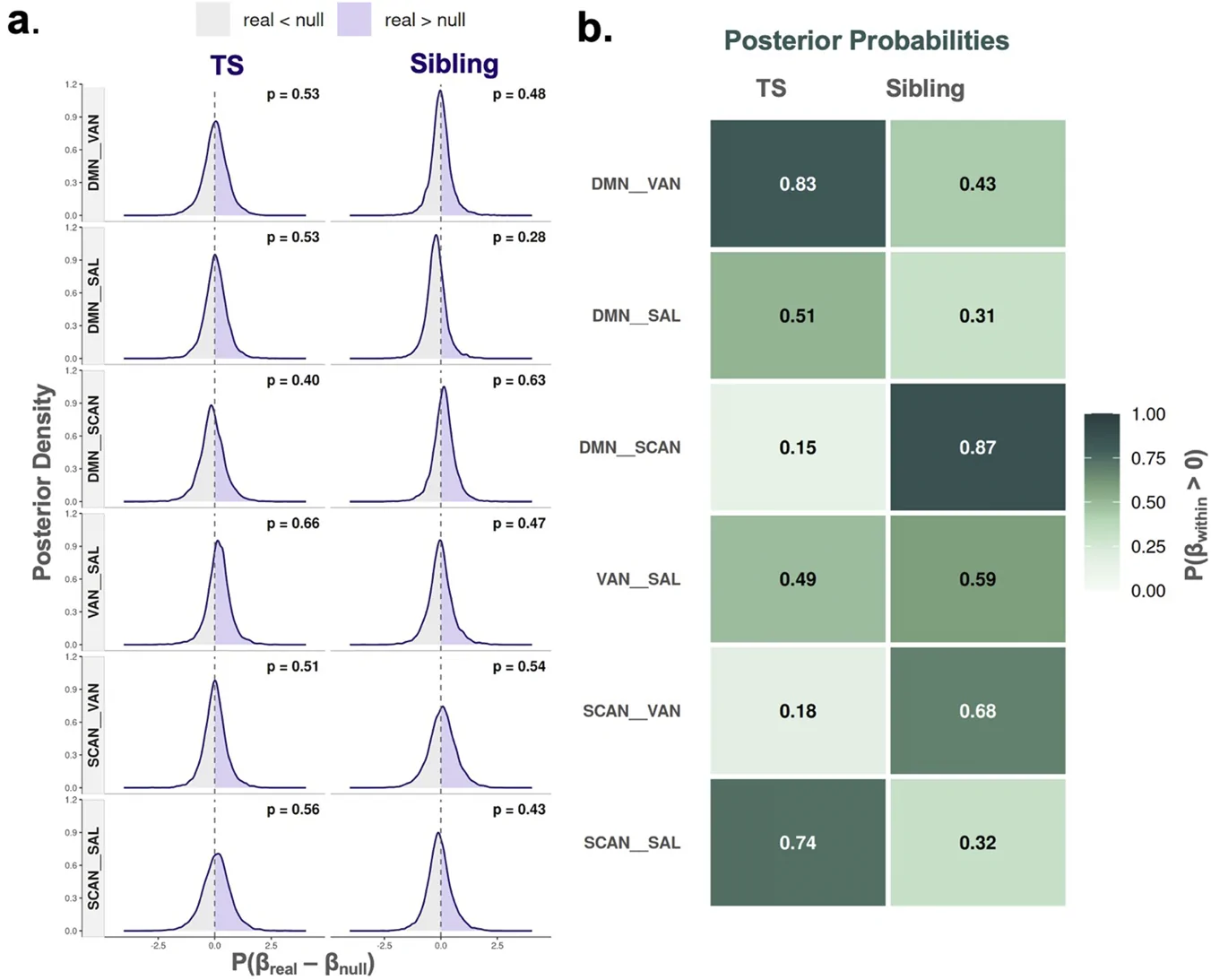
Opposing brain-heart coupling directions at interoceptive network connections in TS and siblings. **(a)** Posterior distributions of P (β_real_ - β_null_) from Bayesian linear mixed-effects models fit separately in TS and sibling participants for six network pairs (DMN-VAN, DMN-SAL, DMN-SCAN, VAN-SAL, SCAN-VAN, SCAN-SAL). For each connection and participant, the within-subject RMSSD slope from observed data (β_real_) was subtracted from the equivalent slope estimated from within-subject, within-run RMSSD permutations (β_null_), yielding a posterior distribution of signal above chance. Purple shading indicates posterior samples where the observed model exceeded the null (real > null); grey shading indicates the reverse. Annotations report P (β_real_ > β_null_), the posterior probability that brain-heart coupling exceeds the shuffle baseline. Both RMSSD and FC were standardized prior to modeling. **(b)** Posterior probability that the within-subject RMSSD slope is positive, P (β_within_ > 0), for each network connection and participant. Darker shading indicates stronger evidence for a positive RMSSD-FC association. The DMN-VAN connection shows the highest probability in the TS participants (P (βwithin > 0) = 0.83), while the unaffected siblings show stronger positive coupling in DMN-SCAN (0.87) and SCAN-VAN (0.68), suggesting divergent interoceptive network organization between individuals with and without TS. Abbreviations: TS, Tourette syndrome; DMN, default mode network; VAN, ventral attention network; SAL, salience network; SCAN, somato-cognitive action network; RMSSD, root mean square of successive differences; FC, functional connectivity.

### 3.5 RMSSD associates with stronger SCAN-AMN but weaker FPN-anchored connectivity in TS

Target Analysis 2 models revealed differing directional dissociation at the SCAN-AMN connection between TS and sibling participants, alongside a pattern of broadly negative RMSSD associations with FPN-anchored connectivity specifically in TS participants (Figure 5; Supplementary Table S2). At the SCAN-AMN, the TS participants showed a positive directional tendency (β = 0.27, 95% CrI [−0.59, 1.02], P (β > 0) = 0.83), whereas their unaffected siblings showed a negative directional tendency in the opposite direction (β = −0.24, P (β > 0) = 0.16). A weaker but directionally consistent reversal was observed at DMN-AMN, where the TS participants again showed a negative tendency (P (β > 0) = 0.32) and the siblings a positive tendency (P (β > 0) = 0.64). Within the FPN-anchored connections, TS participants showed negative directional tendencies at both FPN-AMN (β = −0.21, P (β > 0) = 0.27) and FPN-SCAN (P (β > 0) = 0.38), with FPN-AMN real-data slopes falling notably below the null distribution (P (β_real_ > β_null_) = 0.37). Siblings showed near-null results at these between-network connections. DMN-FPN showed a modest positive tendency in both (TS: P (β > 0) = 0.64; Sibling: P (β > 0) = 0.51) without meaningful differentiation between them. The DMN-SCAN connection, shared across both target analyses, reproduced the same between-participant reversed pattern observed previously. Taken together, Target Analysis 2 results suggest that in TS participants, higher parasympathetic variability co-occurs with stronger SCAN-AMN coupling but weaker connectivity across FPN-anchored connections–a pattern consistent with higher pulse-derived RMSSD being associated with stronger SCAN-AMN coupling while showing weaker, decoupled associations with the FPN executive control circuit that would ordinarily be engaged alongside it. Siblings showed the opposite direction at SCAN-AMN and no evidence of FPN disengagement. As in Target Analysis 1, credible intervals were wide for all between-network connections, and results represent directional trends in a small-sample study.

**FIGURE 5:**
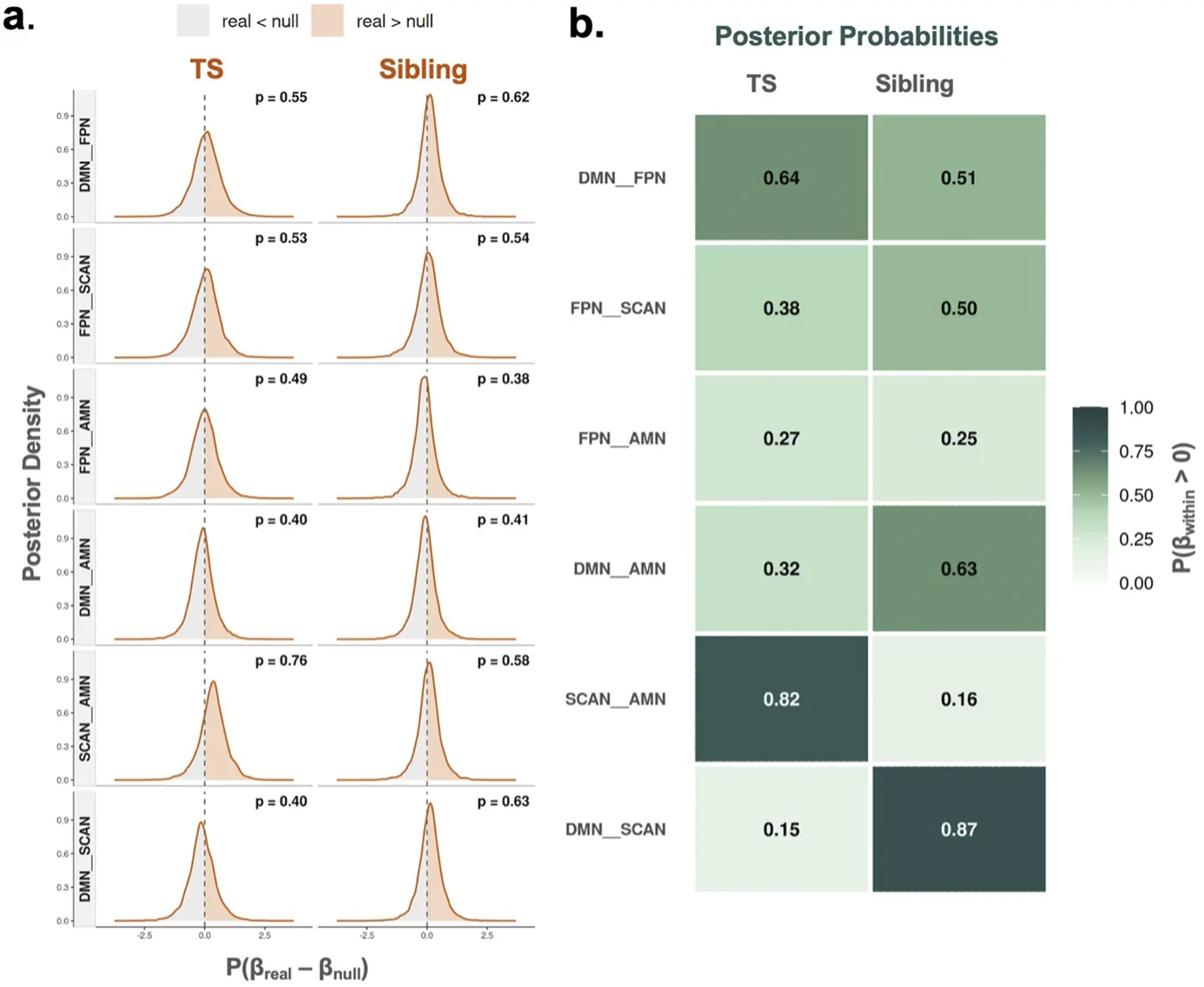
TS-specific SCAN-AMN coupling and FPN disengagement under higher autonomic variability. **(a)** Posterior distributions of P (β_real_ - β_null_) from Bayesian linear mixed-effects models fit separately in TS and sibling participants for six network pairs (DMN-FPN, FPN-SCAN, FPN-AMN, DMN-AMN, SCAN-AMN, DMN-SCAN). For each connection and participant, the within-subject RMSSD slope from observed data (β_real_) was subtracted from the equivalent slope estimated from within-subject, within-run RMSSD permutations (β_null_), yielding a posterior distribution of signal above chance. Orange shading indicates posterior samples where the observed model exceeded the null (real > null); grey shading indicates the reverse. Annotations report P (β_real_ > β_null_), the posterior probability that brain-heart coupling exceeds the shuffle baseline. Both RMSSD and FC were standardized prior to modeling. **(b)** Posterior probability that the within-subject RMSSD slope is positive, P (β_within_ > 0), for each network connection and participant. Darker shading indicates stronger evidence for a positive RMSSD-FC association. SCAN-AMN shows the strongest positive coupling in the TS participants (P (βwithin > 0) = 0.82), consistent with TS-specific modulation of connectivity between the action mode and somato-cognitive networks by cardiac autonomic activity. In contrast, siblings show stronger evidence for positive coupling along DMN-SCAN (0.87) and DMN-AMN (0.63), suggesting that inhibitory control network organization is differentially linked to HRV between individuals with and without TS. Abbreviations: TS, Tourette syndrome; SCAN, somato-cognitive action network; AMN, action-mode network; FPN, frontoparietal network; DMN, default mode network; RMSSD, root mean square of successive differences; FC, functional connectivity.

## 4 Discussion

The present study examined brain-heart coupling in TS across two theoretically motivated functional network analyses in two discordant sibling pairs, using a PFM approach that enabled individualized characterization of large-scale network organization with concurrent heart rate measurement. Based on neurobiological models linking autonomic state to urge generation and inhibitory control problems in TS, we predicted that brain-heart coupling would differ between TS and sibling participants and that TS-specific patterns would be most evident in connections linking internal-state and attentional systems within Target Analysis 1 and action-mode and somato-cognitive systems within Target Analysis 2. Consistent with our predictions, we observed differing directional patterns of brain-heart coupling between the participants with TS and their unaffected siblings across both targeted analyses–a pattern that was not driven by overlapping network connections, as the two analyses shared only the DMN-SCAN connection, and the between-participant dissociation was independently observed across the remaining connections within each analysis. Rather than suggesting uniformly stronger or weaker brain-heart interactions in TS, the directional dissociations observed in this sample set up a hypothesis that autonomic state relates to large-scale network coordination in qualitatively different ways in TS. This is consistent with a broader view of cardiac-autonomic regulation as distributed across a central autonomic network rather than localized to any single network such as the DMN (Beissner et al., 2013; Valenza et al., 2019), suggesting that TS-related reorganization may involve shifts in how this distributed network couples to cardiac-autonomic signals. Support for this hypothesis in a larger sample would have implications for understanding how internal physiological signals are integrated with attentional, action, and regulatory systems in the disorder.

The present study also contributes to the growing recognition that individual differences in brain-heart organization are scientifically meaningful and worth characterizing systematically. The opposing coupling directions observed here suggest that autonomic-neural coupling may vary in ways that carry clinically relevant information, and that group average approaches may obscure this variation. Small-N intensive study designs have demonstrated their value for revealing such individual level structure across fields, from precision neuroimaging (Poldrack et al., 2015; Gordon et al., 2017b; Contijoch et al., 2020; Ali and Lynn, 2026). In this sense, the present study is less a definitive characterization of brain-heart coupling in TS than a demonstration that such coupling is worth measuring individually, and that its directional properties warrants investigation in larger samples.

Within the interoceptive and urge awareness analysis, the most consistent between-participant difference emerged at the DMN-VAN/LANG connection, where the TS individuals showed a positive association between RMSSD and functional connectivity that was absent in their siblings. The DMN supports internally oriented cognition and the representation of bodily and affective states (Buckner et al., 2008), while the VAN/LANG supports stimulus-driven attentional reorienting to salient internal and external events (Corbetta and Shulman, 2002; Corbetta et al., 2008). This network has traditionally been characterized as a ventral attention network based on its role in stimulus-driven reorienting, though recent evidence suggests it may be more accurately characterized as a language network, given its overlap with classic language regions including inferior frontal gyrus and superior temporal cortex (Fedorenko et al., 2024); we retain the VAN/LANG label here to reflect this ongoing debate. A positive RMSSD-DMN-VAN/LANG coupling in TS participants suggests that higher parasympathetic tone is associated with stronger integration between internal state representations and attentional reorienting systems–a pattern consistent with the heightened internal sensations (i.e., premonitory urges) in TS, in which internal bodily signals may more readily capture attentional resources as parasympathetic activity (RMSSD) increases. A directionally similar pattern was observed at SCAN-SAL in the individuals with TS, suggesting that RMSSD also relates to stronger coupling between somato-cognitive action circuitry and salience detection circuitry, potentially reflecting the link between arousal state and the readiness to act on urge-related signals.

Critically, the directional pattern was reversed at DMN-SCAN and SCAN-VAN, where siblings showed consistently positive RMSSD-FC associations while TS participants showed negative tendencies. In siblings, higher autonomic variability was associated with stronger coupling along these connections, suggesting that parasympathetic tone supports more integrated coordination between internal state and somato-cognitive action circuitry–at least in the present sample. This directional tendency was not observed in the participants with TS, raising the possibility that the relationship between pulse-derived RMSSD and coordination across these networks differs between the two sibling groups, though replication in larger samples is needed. Rather than indicating uniformly stronger or weaker brain-heart interactions, these observations are consistent with contemporary neurovisceral integration frameworks in which cardiac autonomic regulation emerges from interactions across a distributed central autonomic network supporting flexible, context-sensitive behavior (Thayer and Lane, 2000; Critchley and Harrison, 2013; Valenza et al., 2019), with TS potentially representing a disorder in which this coordination is reorganized instead of diminished.

Within the inhibitory control and action regulation analysis, the SCAN-AMN connection showed differing directional tendencies between the participants with TS and their unaffected siblings. Specifically, the TS participants showed a stronger positive RMSSD-FC association at this connection while siblings showed a reliably negative tendency in the opposite direction. The AMN supports sustained task control, error monitoring, and physiological regulation, and is strongly connected to the SCAN for whole-body action planning and physiological control (Gordon et al., 2023; Dosenbach et al., 2025). A positive RMSSD-SCAN-AMN coupling in TS participants suggests that as autonomic variability increases, the interface between somato-cognitive action circuitry and action-mode circuitry becomes more tightly coupled, consistent with higher RMSSD being associated with stronger motor-preparatory and action-monitoring circuitry coupling, a pattern that may facilitate or reflect tic generation rather than its suppression, though the present correlational design cannot adjudicate between these possibilities.

In contrast to the SCAN-AMN pattern, TS participants showed negative directional tendencies at FPN-anchored connections, particularly FPN-AMN and FPN-SCAN. This pattern suggests that as autonomic variability increases in TS, connectivity between frontoparietal executive control systems and both AMN and SCAN is reduced rather than increased. The FPN supports top-down regulatory control over motor output and goal-directed behavior (Marek and Dosenbach, 2018), and its apparent weaker coupling during periods of higher RMSSD in TS individuals may relate to reduced executive modulation of action-mode and somato-cognitive circuitry when parasympathetic variability is elevated. Siblings showed no comparable FPN disengagement pattern, suggesting this may be a TS-specific feature of brain-heart organization. Taken together, the Target Analysis 2 results suggest that, in this sample, higher RMSSD showed directional association with stronger SCAN-AMN coupling and weaker FPN-anchored coupling in TS participants–a pattern that may motivate future studies using task-based designs or tic-event monitoring to test whether this dissociation relates to difficulty suppressing tics during states of elevated parasympathetic variability. Although the present study was not designed to assess autonomic dysfunction *per se*, recent work has similarly highlighted the potential utility of HRV measures in adolescents with tic disorders, where altered HRV was associated with quality of life (Mok et al., 2025). Collectively, these findings support further investigation of pulse-derived RMSSD as one component of individualized physiological phenotyping in TS.

The DMN-SCAN connection was included in both targeted analyses by design, serving as a cross-analysis anchor; its posterior estimates are therefore identical across the two analyses, as they derive from a single model. In both TS and sibling participants, this connection showed the most pronounced between-participant dissociation of any connection examined: the unaffected sibling demonstrated a strong positive tendency, while the TS participant showed a consistently negative tendency. This pattern suggests that the relationship between autonomic state and DMN-SCAN coupling may represent a fundamental difference in brain-heart organization between TS and their unaffected siblings in our sample, rather than an analysis-specific artifact. In siblings, higher autonomic variability was reliably associated with stronger integration between internally oriented and somato-cognitive action circuitry, a pattern consistent with parasympathetic tone supporting the flexible integration of internal state signals with action-related circuitry. In TS individuals, this integration appears absent or reversed, suggesting a fundamental reorganization of how autonomic state interfaces with the brain systems that translate internal representations into behavioral output.

The discordant sibling design provides an important interpretive lens for these findings. TS is strongly familial and heritable, and unaffected siblings may share aspects of neurodevelopmental liability even in the absence of clinical tic expression (Pauls et al., 2014; Mataix-Cols et al., 2015; Darrow et al., 2017; Sidiropoulou et al., 2025). By comparing individuals with TS to siblings who share familial background and environment but do not express tics, the design is intended to reduce, though not fully eliminate, the contribution of shared genetic or environmental factors to observed differences; the residual clinical heterogeneity means the present design cannot fully isolate TS-specific effects, and observed differences should be interpreted as hypothesis-generating. The consistent directional dissociations observed here–particularly at DMN-VAN, SCAN-AMN, and DMN-SCAN–therefore might suggest that altered brain-heart coupling is a feature of TS specifically in our sample, emerging in the context of tic expression against a shared familial background. Whether these differences reflect the direct consequences of tic-related neural activity, the broader neurobiological context in which tics arise, or some combination of both remains an open question that future work with larger family-based samples and dimensional measures of tic severity could directly address.

Several limitations should be considered when interpreting these findings. First, the study included only two sibling pairs discordant for TS, which limits generalizability and the ability to draw population-level inferences. Although the dense-sampling design provided many repeated observations within individuals, all findings should be regarded as preliminary and directional, requiring replication in larger samples. Notably, both targeted analyses include DMN and SCAN as constituent networks, meaning that connections within each analysis are not fully independent, shared network nodes contribute to connectivity estimates across both analyses, which should be considered when interpreting the degree of convergence between them. Our sample was also clinically and demographically heterogeneous in ways that could contribute to the observed patterns independent of a TS diagnosis. One participant (PT105) carried co-morbid diagnoses of ADHD, generalized anxiety disorder, and OCD, while her unaffected sibling (PT106) carried ADHD and anxiety; the second pair was diagnostically cleaner (PT100: TS only; PT111: no diagnoses) but differed in age (10 vs. 13) and useable data quantity (Table 1; Supplementary Table S3). Given evidence that HRV changes with age across childhood and adolescence (Gąsior et al., 2018; Goto et al., 1997), and that ADHD and anxiety are independently associated with altered autonomic regulation, we cannot rule out comorbidity or age-related maturation contributing to some of the observed associations. Importantly, approximately 80% of children with TS have one or more comorbid psychiatric disorder (Hirschtritt et al., 2015), and studies of TS therefore do not typically exclude participants with comorbid conditions because doing so would reduce the generalizability of findings to the broader TS population. At the same time, the presence of these comorbidities complicates interpretation of disorder-specific mechanisms in small samples such as the present study. Accordingly, the current findings should be interpreted as preliminary and hypothesis-generating rather than evidence of TS-specific brain-heart coupling.

Second, the duration of usable PPG recordings was shorter than the full duration of the corresponding fMRI runs, reducing the number of analyzable windows and potentially limiting sensitivity. Because our sliding windows overlapped (90-s windows, 45-s steps), successive window-level RMSSD and connectivity estimates are not statistically independent. To address this more directly, we used a circular-shift null model that disrupts temporal correspondence between RMSSD and connectivity while preserving the observed RMSSD values and their within-run temporal autocorrelation structure. Even so, this approach does not fully resolve all dependence introduced by overlapping windows or the small number of participants, and alternative conservative approaches such as phase randomization remain important directions for future methodological work in larger samples.

In addition, RMSSD was used as the primary autonomic measure, which emphasizes short-timescale parasympathetic variability but does not capture the full range of cardiorespiratory dynamics. Because RMSSD was derived from PPG rather than ECG, it represents a pulse-derived estimate of cardiac vagal activity. Although PPG-derived RMSSD demonstrates acceptable agreement with ECG-derived RMSSD under resting conditions, the two measures are not physiologically identical because peripheral pulse recordings are additionally influenced by vascular dynamics and pulse arrival time (Dewig et al., 2024; Plews et al., 2017). Future studies incorporating simultaneous ECG and PPG recordings could further characterize the correspondence between these measures in TS. Respiratory belt recordings were not feasible given that youth with TS frequently experience sensory hypersensitivities that increase intolerance to physical contact and tactile stimulation (Isaacs and Riordan, 2020). Because respiratory contributions were not explicitly modeled during neuroimaging preprocessing, some portion of the observed RMSSD variance may reflect respiratory sinus arrhythmia and/or respiration-related contributions to the BOLD signal. Future studies incorporating simultaneous respiratory monitoring in samples where this is feasible would allow more precise decomposition of cardiac and respiratory components of HRV, enabling cleaner estimates of autonomic-neural coupling in TS.

Moreover, tic events were not independently annotated during scanning; only overall head motion was monitored via FIRMM. As such, we cannot directly link specific tic occurrences to concurrent connectivity or RMSSD fluctuations, and cannot rule out that tic-related motion or transient physiological changes contributed to the observed brain-heart coupling estimates. Third, the present analyses were restricted to *a priori* network pairs during resting state–a deliberate methodological choice that prioritized theoretical specificity, given the small sample size. Finally, our analyses were restricted to cortical functional networks. Subcortical structures central to TS neurobiology including the basal ganglia and thalamus were not included and represent an important direction for future work, particularly given their role in both tic expression and autonomic regulation (Benarroch, 1993; Critchley, 2005).

Future work can extend this approach by integrating PFM with concurrent task-based paradigms (such as voluntary tic suppression or interoceptive attention tasks) and multimodal physiological recordings to more directly characterize the neural mechanisms through which autonomic state shapes tic-related processing in TS. Recent evidence that dynamic functional network organization differs in children with TS (Wang et al., 2026) further supports extending these approaches by integrating individualized network mapping with concurrent physiological recordings. Moving beyond session-bound physiological recordings toward continuous monitoring using wearable PPG devices would also enable more ecologically valid characterization of brain–heart coupling across the daily contexts in which tics and premonitory urges naturally occur. Integrating such wearable-derived measures with periodic neuroimaging in future clinical trials might provide a richer characterization of how autonomic state fluctuates across contexts relevant to tic expression, and whether brain-heart coupling features identified in the scanner generalize to naturalistic settings.

## 5 Conclusion

In summary, this study used PFM and concurrent pulse-derived autonomic measures to characterize brain-heart coupling across interoceptive and inhibitory control network analyses in two densely sampled youth with TS and their unaffected siblings. We observed qualitatively distinct patterns of autonomic-neural coupling between TS and sibling participants, suggesting that autonomic state relates to large-scale network coordination differently in these TS individuals. These preliminary findings, motivate future work in larger family-based cohorts combining multimodal physiological recordings, subcortical connectivity measures, and task-based paradigms to establish the generalizability and mechanistic interpretation of these effects. Ultimately, characterizing individual-specific brain-heart coupling profiles in TS may inform more personalized approaches to treatment, particularly interventions targeting internal state awareness and autonomic regulation such as behavioral therapies, biofeedback, or neurofeedback-based methods.

## Data Availability

The original contributions presented in the study are included in the article/Supplementary Material, further inquiries can be directed to the corresponding author.
