# Supplementary material for: Brain-heart coupling in tourette syndrome: a precision functional mapping study of discordant siblings
Source: Front Netw Physiol. 2026 Sep 23;6:1881914. doi: 10.3389/fnetp.2026.1881914 (PMC13642012; doi:10.3389/fnetp.2026.1881914)
Supplement: Supplementary file 1 [file Supplementaryfile1.docx]

***Supplementary Material***

**Supplementary Table 1. Ranked participant-stratified Bayesian within-subject RMSSD–FC slopes across Target Analysis 1 network connections.** Connections among the default mode (DMN), ventral attention (VAN), salience (SAL), and somato-cognitive action (SCAN) networks are ranked separately for TS and sibling participants by P(β > 0), the posterior probability that the standardized within-subject RMSSD slope is positive. For each network connection, the posterior mean within-subject slope (β_within_), its 95% credible interval (CrI), and P(β > 0) are reported. In the TS participants, the strongest positive tendency was observed for DMN–VAN; in siblings, the strongest positive tendency was observed for DMN–SCAN, with opposing between-participant directions apparent across multiple connections. Credible intervals overlapped zero for all connections; results should be interpreted as directional tendencies rather than conclusive effects.

**Supplementary Table 2. Ranked participant-stratified Bayesian within-subject RMSSD-FC slopes across Target Analysis 2 network connections.** Connections among the frontoparietal (FPN), action mode (AMN), somato-cognitive action (SCAN), and default mode (DMN) networks are ranked separately for TS and sibling participants by P(β > 0). For each network connection, the posterior mean within-subject slope (β_within_), its 95% credible interval (CrI), and P(β > 0) are reported. In the TS participants, the strongest positive tendency was observed for SCAN-AMN, while FPN-anchored connections showed broadly negative slopes; in siblings, the pattern at SCAN–AMN was reversed (rank 6, P(β > 0) = 0.163), with the strongest positive tendency observed for DMN–SCAN. Credible intervals overlapped zero for all connections; results should be interpreted as directional tendencies rather than conclusive effects.

^†^ DMN-SCAN is a shared connection appearing in both analyses; values are identical across tables as they are estimated from the same model.

Network connections are ranked for each participant set P(β > 0), the posterior probability that the standardized within-subject RMSSD slope is positive. β_within_ = posterior mean within-subject RMSSD slope on standardized FC; 95% CrI = 95% credible interval. Both RMSSD and FC were standardized by their pooled standard deviations prior to modeling. Credible intervals overlap zero for all connections; results should be interpreted as directional tendencies rather than conclusive effects. Positive β values are shown in **blue**; negative values in **red**.

| **Participant** | **Study Visit** | **Medication Status** | **YGTSS Total Tic Severity** | **CY-BOCS Total Score** | **MASC Total Score** |
| --- | --- | --- | --- | --- | --- |
| PT105 | Clinical assessment | None | 23 | 3 | 44 |
|  | MRI Session 1 | None | 12 | 0 | – |
|  | MRI Session 2 | Ibuprofen | 14 | 0 | – |
|  | MRI Session 3 | None | 14 | 0 | – |
|  | MRI Session 4 | None | 14 | 0 | – |
|  | MRI Session 5 | Antihistamine | 11 | 0 | – |
|  | MRI Session 6 | None | 13 | 0 | – |
| PT106 | Clinical assessment | None | 0 | 0 | 106 |
|  | MRI Session 1 | Adderall 10mg | 0 | 0 | – |
|  | MRI Session 2 | Adderall 10mg | 0 | 0 | – |
|  | MRI Session 3 | Adderall 5mg | 0 | 0 | – |
|  | MRI Session 4 | Adderall 5mg | 0 | 0 | – |
| PT110 | Clinical assessment | None | 10 | 0 | 63 |
|  | MRI Session 1 | None | 15 | 0 | – |
|  | MRI Session 2 | None | 18 | 0 | – |
|  | MRI Session 3 | None | 24 | 0 | – |
|  | MRI Session 4 | None | 19 | 0 | – |
| PT111 | Clinical assessment | None | 0 | 0 | 41 |
|  | MRI Session 1 | None | 0 | 0 | – |
|  | MRI Session 2 | None | 0 | 0 | – |
|  | MRI Session 3 | None | 0 | 0 | – |
|  | MRI Session 4 | None | 0 | 0 | – |

**Supplementary Table S3. Medication status and longitudinal clinical measures across study visits.** MASC scores were collected only during the baseline clinical assessment; therefore, MASC values are reported only for the clinical assessment visit. An em dash (—) indicates that the measure was not collected at that visit.

*Abbreviations: YGTSS, Yale Global Tic Severity Scale; CY-BOCS, Children's Yale-Brown Obsessive Compulsive Scale, MASC, Multidimensional Anxiety Scale for Children.*

**Supplementary Fig. S1 | Sliding-window brain–heart coupling pipeline for all participants.** Representative examples of the sliding-window analysis for each participant (PT105, PT106, PT110, and PT111). For each participant, the upper panel (**a, c, e, g**) shows sliding-window functional connectivity (FC) estimation between the selected network pair using overlapping 90-second windows with 45-second steps (dashed lines). FC was computed as the Pearson correlation between the mean BOLD time series of the two networks within each window (left), with the resulting window-specific FC values shown at right. The corresponding lower panel (**b, d, f, h**) shows inter-beat interval (IBI) time series used to estimate heart rate variability (HRV; RMSSD) within the same sliding windows (left), with the corresponding window-specific RMSSD values shown at right. Shaded regions indicate the analysis windows used for paired FC and HRV estimation.
